# Characterization of Different Copovidone Grades as Carrier Materials in Hot Melt Extrusion of Amorphous Solid Dispersions

**DOI:** 10.3390/pharmaceutics17091138

**Published:** 2025-08-30

**Authors:** Marvin Daalmann, Vincent Kimmel, Christian Muehlenfeld, Markus Thommes, Judith Winck

**Affiliations:** 1 Laboratory of Solids Process Engineering, Department of Biochemical and Chemical Engineering, TU Dortmund University, Emil-Figge-Str. 68, 44227 Dortmund, Germany; marvin.daalmann@tu-dortmund.de (M.D.); vincent.kimmel@tu-dortmund.de (V.K.); markus.thommes@tu-dortmund.de (M.T.); 2 INVITE GmbH, Drug Delivery Innovation Center, Chempark Building W32, Otto-Bayer-Str. 32, 51061 Cologne, Germany; 3 Ashland Life Sciences R&D, Paul-Thomas-Str. 56, 40599 Düsseldorf, Germany; cmuehlenfeld@ashland.com

**Keywords:** copovidone, extrusion, solid dispersion, solubility, degradation

## Abstract

**Background/Objectives:** Copovidone (polyvinylpyrrolidone-vinyl acetate copolymer, PVP/VA) is a widely used pharmaceutical excipient with various applications in drug formulation. In hot melt extrusion (HME), PVP/VA is an approved carrier material for the production of amorphous solid dispersions (ASDs) by embedding drugs on a molecular level. This study investigates the properties and processability of two copovidone grades—Plasdone™ S-630 (PS-630) and the novel Plasdone™ S-630 Ultra (PS-630U)—to assess their suitability as ASD carrier materials. **Methods:** The thermal and physicochemical characteristics of both polymers were evaluated, focusing on glass transition temperature and polymer melt rheology. The process performance in HME was investigated on small-scale as well as in production-scale extrusion. The two model drugs itraconazole and griseofulvin were used to examine drug dissolution and degradation during HME via in-line UV-vis spectroscopy. **Results:** When comparing both polymers, PS-630U offers various advantages due to the improved powder feeding behavior and reduced yellowing of extruded products while maintaining similar melt properties and drug compatibility compared to PS-630. **Conclusions:** These findings support the use of PS-630U as an optimized copovidone grade for ASD manufacturing, facilitating improved processing characteristics and best product qualities without the requirement of significant formulation adjustments.

## 1. Introduction

Copovidone, also known as polyvinylpyrrolidone-vinyl acetate (PVP/VA), is a versatile pharmaceutical excipient commonly used in drug formulations. It serves several functions, including acting as a binder in tablets [1], a film-forming agent in coatings [2,3,4], and a solubilizer for poorly soluble drugs [5,6,7]. It is a synthetic random copolymer produced by the free radical polymerization of vinylpyrrolidone (VP) and vinyl acetate (VA) with a 6:4 ratio of VP to VA. It is a freely flowing spray-dried powder with a spherical, hollow particle morphology, and was designed to overcome some of the limitations associated with polyvinylpyrrolidone (PVP), such as PVP’s relatively stiff, brittle, and hygroscopic nature. While the brittleness and stiffness of PVP are reflected in a relatively high glass transition temperature (T_g_) of approximately 164 °C for a PVP K30 grade, PVP/VA shows a lower T_g_ of approximately 108–111 °C, with a molecular weight (MW) close to the MW PVP K30. The decrease in the T_g_ of copovidone due to the addition of a comonomer to vinylpyrrolidone improves the copolymer plasticity and flexibility, and thus, PVP/VA has a significantly lower T_g_ and is a lot more flexible and plastic compared to PVP. The combination of good thermoplastic properties and low T_g_ make PVP/VA a suitable polymer for advanced drug delivery techniques such as hot melt extrusion (HME) and the creation of amorphous solid dispersions (ASDs) of poorly water-soluble drugs [8,9,10]. In HME, a powdered mixture composed of drug and at least one thermoplastic polymer, like PVP/VA, is mixed at a molecular level while passing through high-temperature rotating extruder screw elements to form a homogenous matrix. Melting or softening of the matrix occurs due increased heat resulting from either conduction from the extruder housing or viscous dissipation from the shear imparted by rotating screw elements. Due to the mixing and rise in temperature, the drug melts or dissolves into the carrier and distributes to ideally form a homogeneous single phase with the carrier, e.g., an ASD.

The pharmaceutical adaptation of ASDs began in 1992, with to-date more than 50 products being approved by the FDA and manufactured by various methods [11,12,13]. In the early 2000s, advances in the understanding of ASDs have integrated hot melt extrusion (HME) as a central manufacturing process in the pharmaceutical industry, consequently followed by FDA-approved amorphous dispersion products manufactured by HME such as Kaletra^®^ (Ritonavir/Lopinavir with copovidone (PVP/VA)) in 2007, Norvir^®^ (Ritonavir with PVP/VA) and Onmel^®^ (Itraconazole with HPMC) in 2010, and Noxafil^®^ (Posaconazole with hypromellose acetate succinate (HPMCAS)) in 2013. Until today, 16 out of 50 ASD products that received FDA approval are manufactured by HME (Kaletra^®^, Norvir^®^, Onmel^®^, Noxafil^®^, Belsomra^®^, Viekira PAK™/XR™, Technivie^®^, Venclexta™, Mavyret™, Braftovi^®^, Lynparza™, Ubrelvy^®^, Oriahnn™, Qulipta^®^, Alvaiz^®^, Paxlovid^®^). Among those, all products except Onmel (HPMC) and Noxafil (HPMCAS) used PVP/VA as the matrix forming polymer.

In HME, several independent and dependent variables influence the outcome of the product’s final quality [14]: The independent variables are both continuous (screw speed, feed rate, barrel temperature and vent pressure) and discrete (extruder scale, screw configuration, barrel length, die geometry and drug as well as excipient properties). Dependent variables for HME have been identified as the material temperature and melt viscosity, shear rate, torque, pressure, energy input and residence time distribution [14,15]. Accordingly, the properties of the raw materials, especially their thermal properties, determine processing behavior, and thus, potentially also the resulting product quality. Important properties for raw materials in HME include particle size, glass transition temperature (T_g_), melting temperature (T_m_), degradation temperature (T_d_), and melt viscosity as a function of temperature and shear rate [16].

Focusing on the product quality of ASD formulations, the thermal history during HME is essential with regard to drug dissolution in the polymer carrier as well as to degradation [17,18]. In this context, a balance of the material temperature and residence time distribution is crucial. A temperature and residence time that is too low may not be sufficient to dissolve the drug in the polymer, while high temperatures that persist for extended residence times may degrade the materials. As both, the material temperature and the residence time distribution are dependent variables for HME (e.g., influenced by viscosity) [19,20], process conditions must be optimized based on the raw material properties.

Besides the influence of processing, the quality of ASD formulations is determined by the choice of the carrier polymer. Eventually, optimized polymer material properties will enable better processing and best product quality, and thus, knowledge and understanding of the polymer material properties and their significance will facilitate the success of the formulation. The aim of this study was to characterize two different copovidone grades as carrier materials in ASD extrusion, regular Plasdone™ S-630 copovidone (PS-630) and the novel Plasdone™ S-630 ultra copovidone (PS-630U). While both grades are copovidone polymers composed of vinylpyrrolidone and vinyl acetate in a 6:4 ratio, PS-630U is manufactured using an optimized process with tighter control over initiator residues and post-polymerization purification, resulting in significantly lower residual hydrogen peroxide levels (<100 ppm) compared to PS-630 (<400 ppm) as confirmed by both colorimetric and ^1^H qNMR methods [21,22]. This reduction in reactive oxygen species directly correlates with lower oxidative degradation of sensitive APIs such as atorvastatin calcium and quetiapine fumarate in amorphous solid dispersions (ASDs) [21,23].

In addition to peroxide content, PS-630U exhibits a narrower molecular weight distribution and slightly lower average molecular weight (K-value), which contributes to improved powder flowability, reduced torque during hot-melt extrusion (HME), and enhanced feeding behavior [23]. These physical attributes are critical for maintaining consistent processability, especially in high-throughput manufacturing environments.

Furthermore, the lower peroxide content in PS-630U minimizes the formation of N-oxide and hydroxymethyl impurities during both processing and storage, as demonstrated in comparative impurity profiling studies [7]. Interestingly, while PS-630U showed higher free radical generation post-HME, its overall chemical stability remained superior, suggesting that peroxide-driven degradation is the dominant pathway for the APIs studied [7].

While previous studies [7,21,22,23] have started to explore the role of optimized PS-630U copovidone in ASDs, particularly in the context of peroxide quantification, impurity formation, and lab-scale extrusion performance, the present study provides a broader and more application-oriented perspective by integrating process and material science to evaluate PS-630U across the full formulation lifecycle. Specifically, it combines rheological modeling (Carreau-WLF), powder flow characterization, and in-line analytical techniques (UV–Vis) to assess not only chemical stability but also process efficiency, feeding behavior, and scalability. Importantly, it incorporates production-scale trials and evaluates practical considerations such as torque reduction and powder bridging, to enable formulators and pharmaceutical manufacturers to make informed decisions about formulation design, excipient selection, process optimization, and manufacturing strategy. Therefore, raw material properties of PS-630 novel PS-630U as well as their processability in HME were investigated. The quality of the resulting ASD formulations with the model drugs itraconazole and griseofulvin were assessed through in-line UV-vis spectroscopy based on drug dissolution in the polymer and degradation. Finally, evaluating the differences between the two copovidone grades enabled a recommendation for optimized process management when transitioning manufacturing operations to the novel PS-630U.

## 2. Materials and Methods

### 2.1. Materials

**Figure 1:**
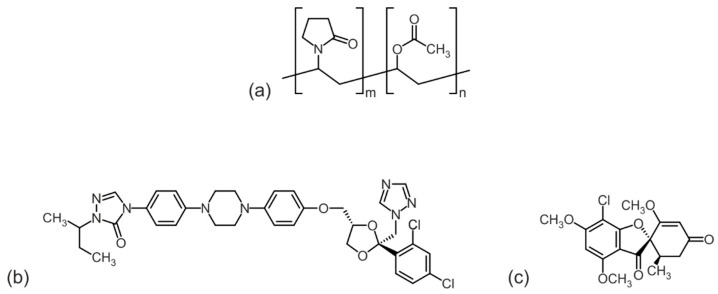
Molecular structure of copovidone (**a**) and the drugs itraconazole (**b**) and griseofulvin (**c**).

Two copovidone grades, Plasdone™ S-630 (PS-630) and Plasdone™ S-630 ultra (PS-630U) (both from Ashland, Wilmington, DE, USA) were characterized and compared with each other. Furthermore, itraconazole (ITR) (BASF SE, Ludwigshafen, Germany) and griseofulvin (GRI) (Hawkins, Roseville, MN, USA) were selected as model drugs for the preparation of ASD formulations (Figure 1). The model drugs were selected to cover a broad range of melting temperatures, with values of 165 °C for itraconazole and 221 °C for griseofulvin [24]. This selection enables the evaluation of ASD formulations with the two different copovidone grades in distinct processing temperature ranges, each associated with different risks of thermal degradation. The melting point of itraconazole is approximately 50 °C above the glass transition temperature of the polymer, placing it within the preferred range for HME [25,26]. In contrast, griseofulvin requires processing at substantially higher temperatures, allowing investigation of the upper processing limit, close to the onset of copovidone thermal degradation [26]. Both model drugs can be molecularly dispersed in copovidone and their use in ASD formulations has been frequently reported. Detailed analyses of the interactions between these drugs and copovidone, as well as the corresponding phase diagrams, have been reported previously [24].

All polymer samples used in this study were sourced internally by Ashland. While no externally supplied control materials were included, the study was designed to objectively assess material performance under standardized conditions. The findings complement existing literature and provide additional insights relevant to pharmaceutical development and manufacturing.

### 2.2. Characterization Methods

#### 2.2.1. Differential Scanning Calorimetry

Differential scanning calorimetry (DSC) was performed using a Q2000 heat flux DSC (TA Instruments, New Castle, DE, USA) that was purged with 50 mL/min nitrogen. For each measurement, between 4 and 5 mg of the sample was placed in an aluminum pan and sealed with a perforated lid. A two-step heating protocol was applied to evaluate the glass transition temperatures. In the first heating cycle, the thermal and mechanical history of the samples were eliminated and remaining moisture was removed. In the second heating cycle, the glass transition temperatures were measured under a heating rate of 20 °C/min.

#### 2.2.2. Rheology

The viscosity measurements were performed using a Haake Mars 60 rheometer (Thermo Fischer Scientific, Karlsruhe, Germany) with a 20 mm plate-plate geometry and a 1 mm gap in oscillation mode. The temperature was varied in a range of 160 to 190 °C. The measurements were conducted within the linear viscoelastic region which was determined using amplitude sweeps. A deformation amplitude of 1% was found to be suitable for all further frequency sweep measurements where the flow curves (complex viscosity over angular frequency) were derived. All measurements were performed in triplicate.

Vacuum compression molding (VCM) was applied as preparation procedure to generate bubble-free samples for precise viscosity measurements. Therefore, a VCM device with a 25 mm cylindrical chamber (MeltPrep GmbH, Graz, Austria) was utilized [27]. The temperature was set to 172 °C for 12 min which is approximately 63 °C above the glass transition temperature as recommended by the manufacturer [28]. The prepared polymer films were stored in a desiccator until the subsequent viscosity measurements in order to avoid water sorption.

#### 2.2.3. Bulk Properties

The bulk ($\rho_{bulk}$) and tapped density ($\rho_{tap}$) of the polymer powders were determined using the tap density tester TD1 (SOTAX AG, Aesch, Switzerland).

(1) $$\rho_{bulk/tap}=\frac{m}{V_{bulk/tap}}$$

The densification of the powders were characterized by calculating the Carr index (CI).

(2) $$CI=\frac{\rho_{tap}-\rho_{bulk}}{\rho_{tap}}$$

Furthermore, the powder flow were evaluated based on measuring the angle of repose (α) using a PF1 powder flow tester (SOTAX AG, Aesch, Switzerland). The angle of repose was geometrically determined from the height (h) and the radius (r) of the powder bed.

(3) $$tan(\alpha )=\frac{h}{r}$$

#### 2.2.4. Moisture Content

Karl-Fischer Titration was applied to determine the water content of the powder samples. Therefore, a Titrando (Deutsche METROHM GmbH & Co. KG, Filderstadt, Germany) was utilized.

### 2.3. Extrusion

#### 2.3.1. Small Scale Extrusion

**Figure 2:**
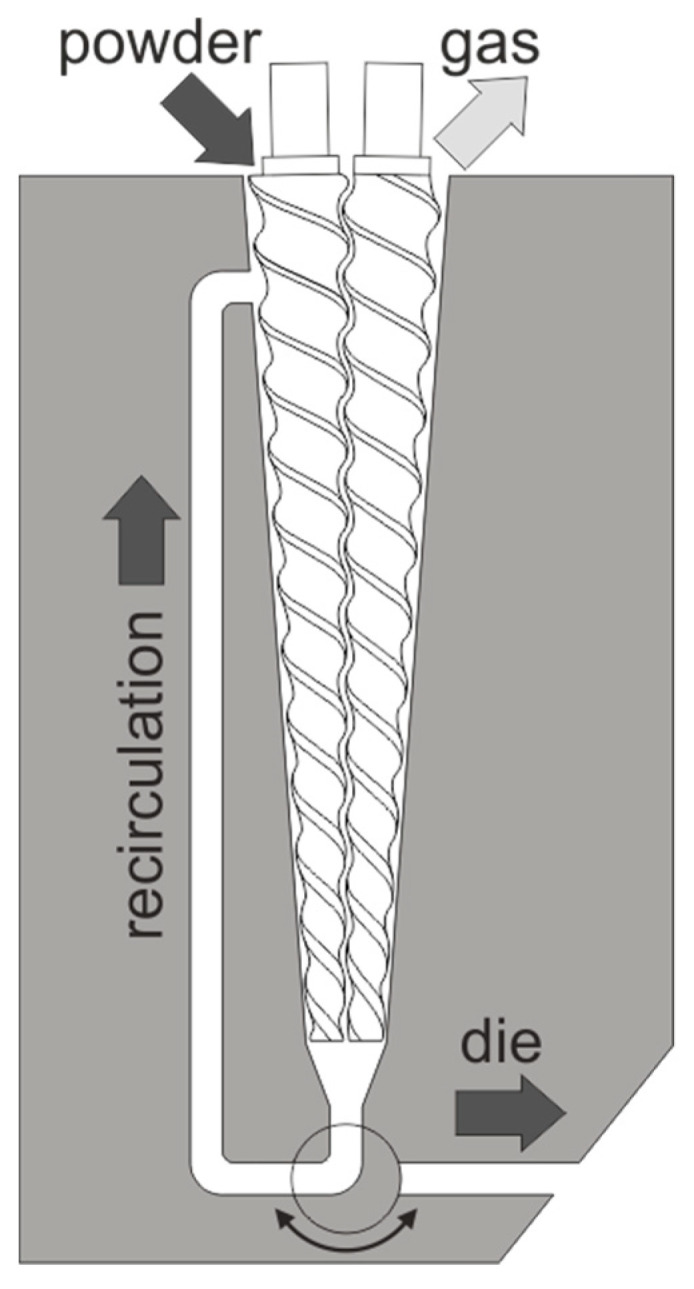
Configuration of the microcompounder for small scale extrusion.

Small scale extrusion was conducted using a DSM Xplore microcompounder (Xplore Instruments BV, Sittard, Netherlands), featuring two intermeshing conical screws, a 5 mL capacity, and a 3 mm die diameter (Figure 2). The screw speed was kept constant at 50 rpm throughout the study. Each run utilized 2.5 g of formulation fed into the extruder via an air-cooled hopper to prevent powder adhesion. The material residence time was controlled by operating the extruder in recirculation mode, achieved by adjusting the valve between the screw tips and the die to the recirculation channel. Material temperature was regulated by six heating elements in the extruder barrel.

A systematic parameter study was conducted for both drugs across three individual drug weight fractions. For each drug, seven temperatures were selected, and extrusion was performed at three residence times (1 min, 3 min, and 10 min) for each formulation and temperature to explore their influence.

#### 2.3.2. Production Scale Extrusion

The production scale extrusion experiments were carried out in a co-rotating twin screw extruder (ZSE 27 MAXX, Leistritz, Nuremberg, Germany) with a 28.3 mm diameter and a length of 40 D. A heated extrusion die with a 3 mm diameter and 11.7 mm length was chosen. The powder material was metered by a loss-in-weight feeder (K-Tron K-ML-SFS-KT20, Coperion, Niederlenz, Switzerland) into the extruder feeding port.

**Figure 3:**
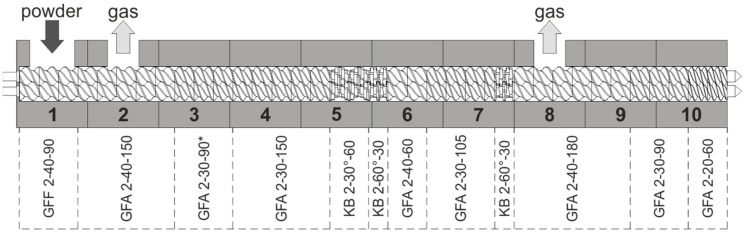
Screw and barrel configuration for production scale extrusion.

The extruder screw was modularly configured with two kneading zones, derived from commonly used extruder setups for hot melt extrusion of ASD formulations (Figure 3). The rotational speed was kept constant at 200 rpm. The temperature profile was altered in a range from 150 °C to 190 °C which means that the barrels 5 to 10 were set to the desired temperature, while the temperature of barrels 1 to 4 were kept constant at a lower temperature (25 °C, 25 °C, 25 °C, 100 °C, respectively) to guarantee sufficient powder transport before plastification in the first kneading section. Resulting extrusion parameters were measured when the torque and the pressure at the die had reached a constant value. The die pressure was measured by a pressure transducer (KE1-7-M-B35D-1-4-D-S-P-E, Gefran, Provagilo d’Iseo, Italy). The melt temperature at the die was measured with an IR-camera (TESTO 875, Testo SE & Co. KGaA, Lenzkirch, Germany) using a pre-determined emission coefficient of 0.93 and a reflecting temperature of 60 °C.

### 2.4. In-Line UV-Vis Spectroscopy

The UV-Vis spectroscopy was conducted using an Inspectro X spectrophotometer (ColVisTec, Berlin, Germany) in transmission mode, covering the range of 224 to 820 nm with a sampling rate of 1 Hz. Each exposure involved two xenon lamp flashes lasting 10 ms. Spectra were calibrated using the pure polymer as a blank reference. Special ColVisTec probes with single fibers were integrated, distinct from standard TPMP probes. These probes measure small material quantities in the small die channel as already qualified in a previous work [17]. The probes were affixed to a spacer sleeve with union nuts, maintaining a specific probe distance. The flow channel matched the 3 mm diameter of the extruder die, ensuring uniform flow.

#### 2.4.1. Determination of Drug Dissolution in the Polymer and Degradation

ASD formation during extrusion was assessed via visible range UV-Vis spectra. The dissolution of the drug in the polymer was determined using the lightness (L*) value of the CIELAB color system [29]. L* represents perceptual lightness from black (L* = 0) to white (L* = 100), indicating turbidity [30]. The lightness was given here as a percentage relative to the highest lightness detected for PS-630 in small scale extrusion at 153 °C and a residence time of 1 min. Changes in lightness during extrusion mainly reflect drug dissolution rather than polymer yellowing, as light scattering of undissolved drug crystals reduces transmitted light intensity. The assessment of whether drug crystals are present in the extrudate, or whether a fully amorphous system has formed, using the described light scattering method is known to provide results that are consistent with PXRD measurements [31].

On the other hand, yellowing indicates degradation, which was monitored via the b* value of the CIELAB color system (shift from blue to yellow discoloration). Here, the yellowing was given as a percentage relative to the highest yellowing detected for PS-630 in small scale extrusion at 192 °C and a residence time of 10 min. For both, the L* and b* value*, 10 measurements were carried out, and the mean as well as the standard deviation were determined. ΔE values were not evaluated because they combine changes in L*, a*, and b* CIELAB color values into a single metric, preventing differentiation between drug dissolution (L*) and yellowing (b*), which were intended to be evaluated independently.

#### 2.4.2. Determination of Residence Time Distributions

Quinine-dihydrochloride (Caesar & Loretz, Hilden, Germany) was utilized as tracer for the residence time determination with a fraction of about 10 mg per polymer mass flow of 1 kg/h. The tracer was added as a Dirac-impulse through the hopper of the extruder. The response signal was measured in the die with the inline UV-Vis-spectrophotometer and evaluated at a wavelength (λ) of 315 nm. The transmission (Tr) is expressed as the ratio of transmitted light intensity (I) to the basic light intensity of the polymer ($I_{0}$).

(4) $$Tr=\frac{I\left(\lambda \right)}{I_{0}\left(\lambda \right)}$$

The transmission was converted to an absorbance (A) using the Lambert-Beer law.

(5) $$A\left(\lambda \right)=-{log}_{10}\left(\frac{I\left(\lambda \right)}{I_{0}\left(\lambda \right)}\right)=c_{tracer}\cdot l_{path}\cdot \varepsilon_{\lambda }$$

The residence time density function ($E\left(t\right)$) was derived from the tracer concentration ($c_{tracer}$) over time under constant path length ($l_{path}$) and absorbance coefficient ($\varepsilon_{\lambda }$). Mean residence times ($\bar{t}$) were evaluated based on the residence time density functions.

(6) $$\bar{t}=\int_{0}^{1}t\cdot E\left(t\right)\;dt$$

The integrals were calculated by the trapezoidal method using MATLAB^®^ (The MathWorks, Inc., Natick, MA, USA).

## 3. Results and Discussion

### 3.1. Comparison of Copovidone Raw Material Properties

**Figure 4:**
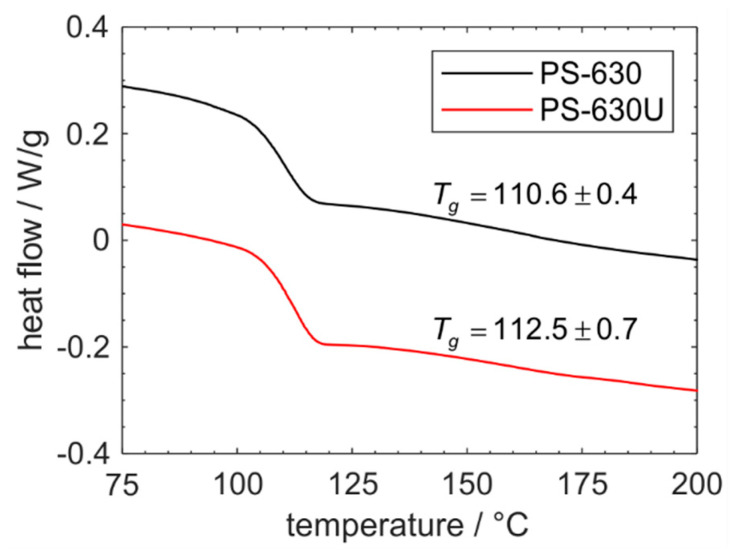
Representative DSC thermograms of PS-630 and PS-630U and glass transition temperatures (*n* = 3).

The two copovidone grades show a glass transition in the DSC thermograms and no additional phase transitions thereafter, indicating that both polymers are fully amorphous (Figure 4). The glass transition temperature of PS-630U (112.5 ± 0.7 °C) is approximately 2 °C higher than that of PS-630 (110.6 ± 0.4 °C). This slightly higher glass transition temperature of PS-630U is also reported in the literature [23]. However, the literature values of the absolute glass transition temperatures show small deviations to this study which is attributed to different heating rates in the measuring procedure.

**Figure 5:**
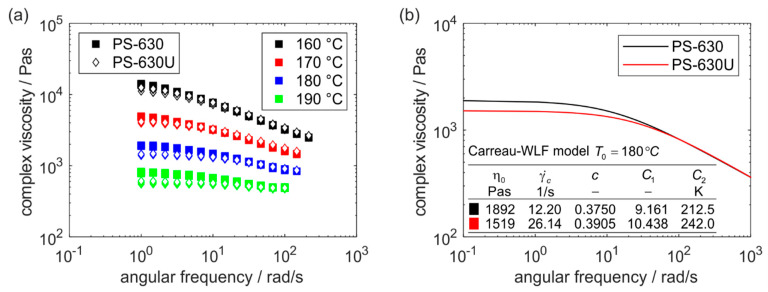
Viscosity curves (*n* = 3) from oscillatory rheometry of PS-630 and PS-630U (**a**) and Carreau-WLF model at a reference temperature of 180 °C according to the time-temperature superposition (**b**).

From the melt rheology, it is evident that the viscosity decreases with increasing angular frequency for both copovidone grades (Figure 5a). This corresponds to the shear-thinning behavior, commonly seen for polymers used in hot melt extrusion. The macromolecules have the highest entropy in the resting state. As shear stresses increase, entropy decreases, requiring less energy to allow polymer chains to slide past each other. Consequently, viscosity decreases with increasing shear rate.

The viscosity data is described by the Carreau model [32]

(7) $$\eta =\frac{\eta_{0}}{{\left(1+\frac{\dot{\gamma }}{{\dot{\gamma }}_{c}}\right)}^{c}}$$

whereby the dynamic viscosity (η) is a function of the shear rate ($\dot{\gamma }$) and the three material-dependent parameters zero-shear viscosity ($\eta_{0}$), critical shear rate (${\dot{\gamma }}_{c}$) and flow exponent (c). According to the time-temperature superposition, the viscosity curves described by the Carreau model are shifted to one master curve at a reference temperature ($T_{0}$) of 180 °C (Figure 5b).

(8) $$\eta =\frac{\eta_{0}a_{T}}{{\left(1+\frac{\dot{\gamma }a_{T}}{{\dot{\gamma }}_{c}}\right)}^{c}}$$

The shift factor $a_{T}$ is expressed using the Williams-Landel-Ferry (WLF) equation [33] which relates the temperature dependence of material properties to a shift in time scales utilizing the universal constants $C_{1}$ and $C_{2}$.

(9) $$log\left(a_{T}\right)=-\frac{C_{1}\left(T-T_{0}\right)}{C_{2}+T-T_{0}}$$

The master curves at 180 °C show a higher viscosity of PS-630 in comparison to PS-630U at low shear rates. As the shear rate increases, both curves increasingly converge and subsequently overlap. The zero-shear viscosity of PS-630U is 20% lower than that of PS-630. In contrast, the critical shear rate of PS-630U is higher and the both polymers show a similar flow exponent, so that the viscosities of PS-630U and PS-630 are the same at angular frequencies of over 40 rad/s. According to the Cox-Merz rule [34] that was already found to be applicable for copovidone [35], the complex viscosities over angular frequency are comparable to the dynamic viscosities over shear rate. Consequently, both polymers will show the same flow behavior when they are strongly sheared in the screw clearances during extrusion. In contrast, the lower shear rates in larger free areas in an extruder result in a lower viscosity of PS-630U during processing. Since the clearances only represent a small part of the free extruder volume, PS-630U requires less energy input during processing. Accordingly, PS-630U can be processed at lower temperatures, while still showing the same flow behavior as PS-630 at a higher temperature. More precisely, the same zero-shear viscosity for PS-630 at 180 °C (1892 Pas) is observed for PS-630U at 177.81 °C.

The powder bulk properties provide information about the powder flow and therefore initial indications regarding the feeding behavior in the extruder. The bulk density of PS-630U is 16% lower than that of PS-630 (Table 1). The tapped density of PS-630U is 18% lower compared to PS-630.

The densification of the polymer powders is evaluated based on the Carr index. The Carr index of 20.13% for PS-630 is higher than that of PS-630U, which is 17.73%. The difference between tapped and bulk density indicates compaction for both polymers, leading to decreased flowability. This is attributed to interparticular interactions between the powder particles. According to European Pharmacopoeia guidelines [36], the powder flow behavior is assessed by means of the Carr index as fair (16–20%) for both polymers. However, PS-630 lies in the transition range to passable flow (21–25%).

Another parameter used to describe flowability is the angle of repose of a powder bed. The angle of repose of 36.28° for PS-630U is approximately 20% higher than that of PS-630, which is 29.17°. The lower and wider the powder bed, the smaller the angle of repose and the better the flowability. Flowability can be considered as fair (36–40°) for PS-630 and excellent (25–30°) for PS-630U according to European Pharmacopoeia guidelines [36]. The higher standard deviation for PS-630 is due to the fact that the powder only flowed out of the funnel through tapping and gentle stirring, making it more challenging to build a reproducible powder bed for measurement.

### 3.2. ASD Formulation in Small Scale Extrusion

The formation of ASDs is evaluated by detecting residual drug crystals in the extrudates that remain undissolved in the polymer during extrusion. For the complete dissolution of the drug and the formation of an amorphous solution, the material temperature must at least reach the solubility temperature of the drug in the polymer. Furthermore, the dissolution of the drug is influenced by the residence time in the extruder. Unlike in a phase diagram in equilibrium, the dissolution process in extrusion is governed by diffusion kinetics [20,24].

**Figure 6:**
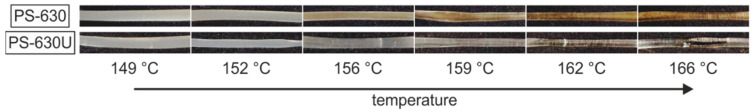
Extrudates of PS-630 (**top**) and PS-630U (**bottom**) at various material processing temperatures with 15 wt% GRI at a residence time of 3 min.

Depending on the melt temperature and residence time, the extrudates show a transition from opaque to transparent appearance, as exemplarily depicted for the GRI formulation with 15 wt% drug in Figure 6. At lower melt temperatures, the extrudates were opaque, signifying the presence of undissolved drug crystals. Above a specific temperature threshold, the appearance shifted from opaque to transparent for all formulations, indicating that the temperature for the respective residence time was sufficient to completely dissolve the drug in the polymer. The visual comparison of the extrudates from PS-630 and PS-630U already suggests that the complete dissolution of the drug is achieved at similar temperatures, as the transition from opaque to transparent appears comparable. However, a stronger yellowing is noticeable for PS-630 compared to PS-630U at higher temperatures. In order to prove this first visual impression, UV-Vis spectroscopic data is evaluated in the following to quantify the ASD formation (Section 3.2) as well as the yellowing (Section 3.3).

In case the drug is not completely dissolved, the remaining crystalline particles scatter the light during UV-Vis spectroscopy, reducing the detected light intensity and hence the lightness L* of the CIELAB color system. The L* value is sensitive to this scattering effect and is therefore a suitable parameter for describing the drug dissolution behavior, eliminating the need for a complete analysis of the spectra.

**Figure 7:**
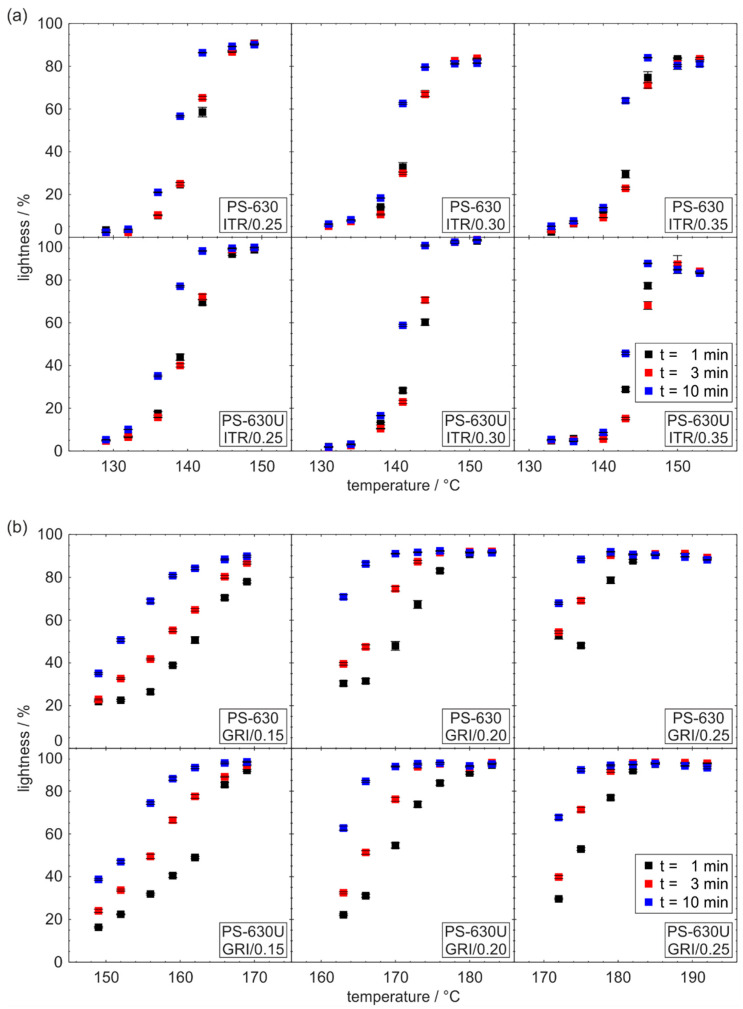
Lightness measured in-line in the die as an indicator of drug dissolution in the polymer for formulations containing itraconazole (**a**) and griseofulvin (**b**) (*n* = 10).

Figure 7 illustrates the relationship between the lightness and extrusion temperature as well as residence time for formulations containing itraconazole (a) and griseofulvin (b). All formulations exhibit a characteristic sigmoidal course, where lightness starts at lower values and increases with temperature until reaching a maximum. This trend is due to the decreasing presence of undissolved drug particles, which scatter light. As a result, the plateau at maximum lightness indicates complete dissolution of the drug in the polymer.

For a given temperature, lightness increases with longer residence times, confirming that more drug particles can be dissolved by applying either higher extrusion temperatures or extended residence times. In general, achieving complete dissolution and transparency at shorter residence times requires higher temperatures. Between the two drugs studied, the impact of residence time on drug dissolution was more pronounced for griseofulvin. This shows that the dissolution of griseofulvin in the carrier polymer is more hindered by kinetic effects than the dissolution of itraconazole. Additionally, the dissolution of griseofulvin requires higher temperatures compared to itraconazole, which is in good accordance with the solubility temperatures stated in phase diagrams of a previous study [24].

When comparing PS-630 and PS-630U, no difference in the dissolution temperature at the plateau with maximum lightness is noticeable. Consequently, the interaction of the studied drugs with both polymers seems to be comparable, and there is no need to adjust the dissolution process when changing from the conventionally used PS-630 to PS-630U, neither in terms of processing temperature nor residence time. However, the lightness range of the measurement points for both polymers is different. For PS-630U, the value at the maximum lightness plateau is a few percent higher across all formulations. This difference is particularly noticeable in the formulations with 25 and 30 wt% itraconazole which is assigned to lower processing temperatures. Accordingly, it is concluded that yellowing and dissolved drug leads to small decrease in lightness due to a differing adsorption behavior of the incoming light. However, the characteristic sigmoidal course of lightness is hardly affected so that no impairments in evaluating the dissolution temperature are to be expected.

### 3.3. Degradation During Small Scale Extrusion

**Figure 8:**
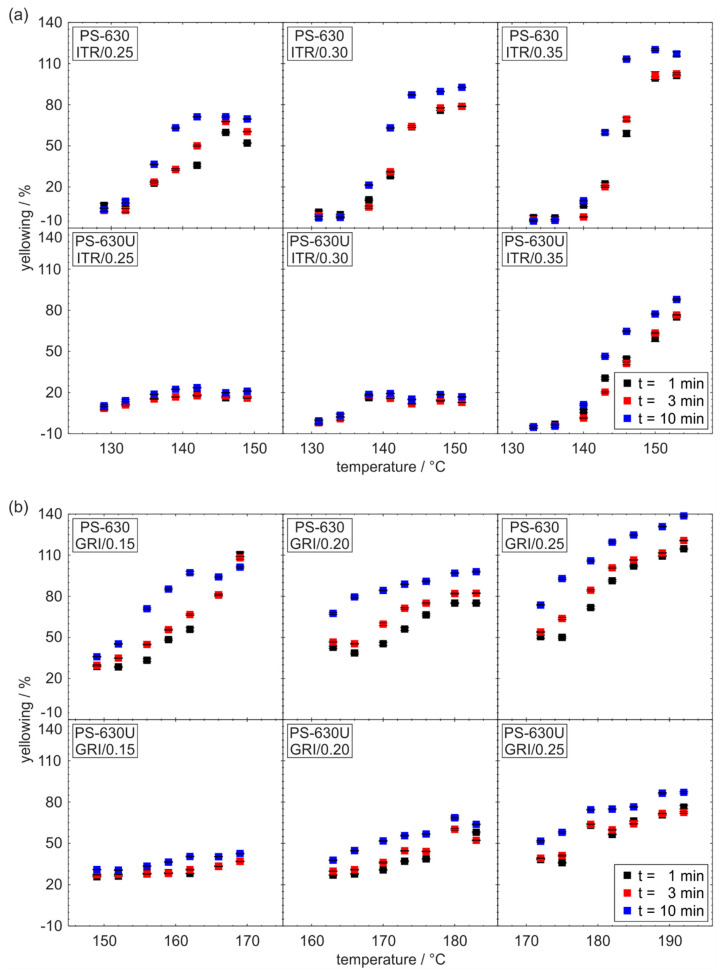
Yellowing measured in-line in the die as an indicator of degradation for formulations containing itraconazole (**a**) and griseofulvin (**b**) (*n* = 10).

The monitoring of degradation processes is essential in HME to produce ASDs of sufficient quality. A yellow to brown discoloration indicates drug and polymer decomposition [31,37]. However. UV-Vis spectroscopy was found to be highly sensitive to these color changes and the subsequent quantitative evaluation of degradation [38]. In Figure 8, the yellowing of extrudates from small scale extrusion is evaluated based on the b* value of the CIELAB color system for formulations containing itraconazole (a) and griseofulvin (b).

In general, the yellowing increases with temperature and residence time. However, large differences in yellowing were observed when comparing both copovidone grades. For the PS-630 formulations, the yellowing is larger than for the PS-630U formulations. Furthermore, the yellowing increases more significantly with the processing temperature for the PS-630 formulations. For a specific formulation, the yellowing between the residence times from 1 to 10 min differs more strongly for the PS-630 formulations. This is explained by the kinetics of degradation processes that follow a certain degradation rate.

If the yellowing is independent from the residence time, this suggests that the color change is more likely due to the active ingredient dissolved in the polymer rather than degradation processes. The PS-630U formulations with low drug load (ITR/0.25, ITR/0.30, GRI/0.15) appear to show little to no degradation accordingly. At higher drug load, degradation was observed for PS-630U, but to a lesser extent than for PS-630. This finding is in good accordance with results from the group of Repka who investigated trace levels of peroxides in PS-630 and PS-630U that caused both degradation and corresponding decreases in purity levels of drugs sensitive to oxidation [21,23]. According to their study, the suitability of PS-630U over PS-630 in developing ASDs was suggested for drugs sensitive to oxidation. Finally, degradation alters both mechanical and chemical properties, thus affecting the quality of the ASD as well.

### 3.4. Application in Production Scale Extrusion

In addition to screening studies in small scale extrusion, the transfer to production scale extrusion is essential to evaluate the processing of the two copovidone grades under production conditions. In this context, the powder flow behavior in the extruder feed section, the processing parameters during extrusion, and the quality of the processed products are of particular interest.

The specific feed load (SFL) is a dimensionless number that describes the barrel load in extrusion. It is calculated from the throughput of the powder ($\dot{m}$), the true density of the polymer ($\rho_{material}$ = 1190 kg/m^3^ [19]), the screw speed (n) and the screw diameter (d) [39].

(10) $$SFL=\frac{\dot{m}/\rho_{material}}{n\cdot d^{3}}$$

A normalized SFL value (${SFL}^{*}$) can be obtained by considering the free cross-sectional area ($A_{free}$ = 0.000491 m^2^) and the pitch ($l_{pitch}$) of the screw in the feeding section, as well as the bulk density ($\rho_{bulk}$) and a slip factor (s) representing the powder flow.

(11) $${SFL}^{*}=\frac{\dot{m}/\rho_{bulk}}{(1-s)\cdot l_{pitch}\cdot A_{free}\cdot n}$$

Here, ${SFL}^{*}$ = 0 corresponds to an empty feeding section, whereas ${SFL}^{*}$ = 1 represents a completely filled feeding section [19]. The maximum SFL value (${SFL}_{max}$) is observed as the developed backlog in the completely filled feeding section when the screw speed is gradually reduced at a constant throughput. In order to evaluate the powder flow of the copovidone grades in the feeding section, the slip factor is determined under consideration of the experimentally measured ${SFL}_{max}$, as well as the correlations from Equations (10) and (11) at ${SFL}^{*}$ = 1.

(12) $${SFL}_{max}=\left(1-s\right)\cdot \frac{\rho_{bulk}}{\rho_{material}}\cdot \frac{A_{free}\cdot l_{pitch}}{d^{3}}$$

${SFL}_{max}$ was found to be 30% lower for PS-630 than for PS-630U, although the bulk density of PS-630 is higher which should lead to a higher ${SFL}_{max}$ in case of an identical powder flow bahavior (Table 2). This proves the enhanced flow behavior of PS-630U as previously assumed based on the lower Carr index and angle of repose (Table 1). When processing PS-630U in HME, the lower slip factor leads to an improved feeding performance and less obstacles with powder bridging in the extruder hopper are expected.

**Figure 9:**
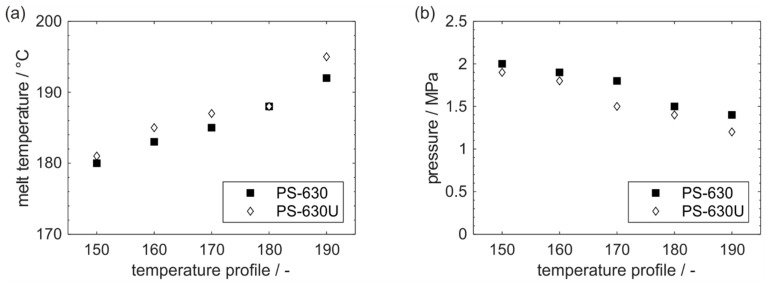
Melt temperature (**a**) and pressure (**b**) in extrusion.

Application studies in production scale were performed by varying the barrel set temperature in 10 °C steps from 150 to 190 °C. The melt temperature measured at the die was in all cases higher than the respective barrel set temperature of the corresponding temperature profile (Figure 9a). This deviation is caused by shear heating due to dissipation which is known to have a large impact on the resulting material temperature rather than an active heating and cooling of the extruder barrels.

When comparing the material temperatures of both copovidone grades, a slightly higher temperature of PS-630U is noticeable at the various temperature profiles. These differences in shear heating are attributed to the individual melt rheology of the polymers and the duration of shearing in the extruder (residence time) [20]. According to the slightly higher material temperatures and lower viscosities of PS-630U, slightly lower pressures were observed at the die (Figure 9b).

**Figure 10:**
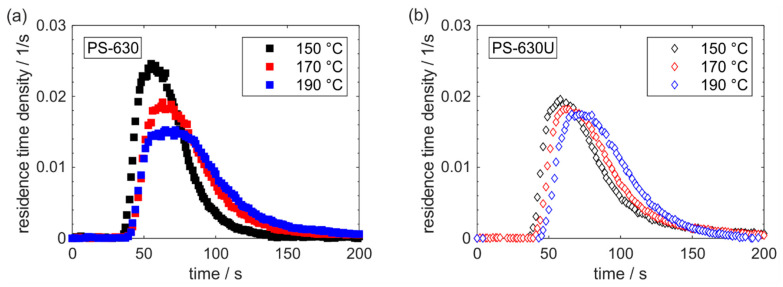
Residence time distribution of PS-630 (**a**) and PS-630U (**b**).

The residence time measurements show the characteristic courses of residence time distributions, commonly known from extrusion (Figure 10). A steep slope during the increase in tracer concentration is followed by a slower decrease, which particularly characterizes the back-mixing in the extruder [40]. Overall, the residence time distributions were shifted to longer times and became wider when increasing the temperature profile to higher temperatures. In comparison of the two copovidone grades, a higher effect of the temperature profile on the residence time distribution was identified for PS-630.

**Figure 11:**
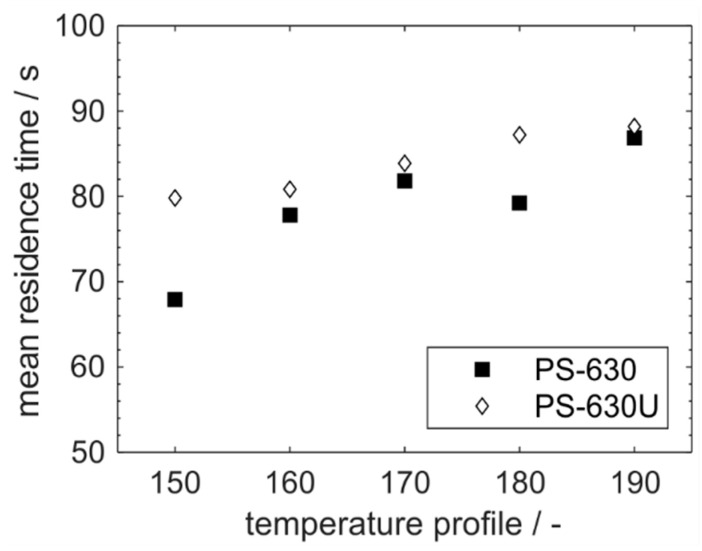
Mean residence time of PS-630 and PS-630U during production scale extrusion.

PS-630U exhibited slightly higher mean residence times than PS-630 (Figure 11). In general, higher mean residence times were observed for lower viscosities, namely for higher temperatures as well as for PS-630U when comparing both copovidone grades. The mean residence time can be correlated with the degree of filling in the extruder by considering that the hydrodynamic residence time in completely filled sections does not change for a constant mass flow. Consequently, the degree of filling increases with decreasing viscosities.

**Figure 12:**
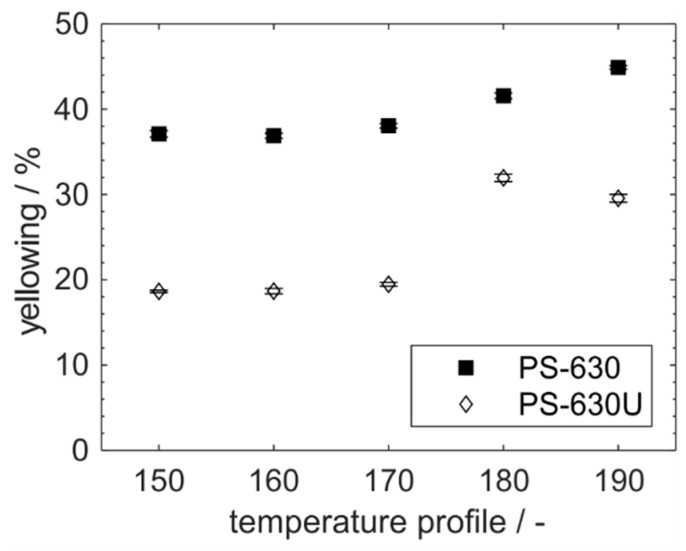
Yellowing of PS-630 and PS-630U during production scale extrusion (*n* = 10).

In addition to Section 3.3, the yellowing of both copovidone grades is also evaluated for the production scale extrusion (Figure 12). A significantly lower yellowing is observed here for PS-630U which is in good accordance with the results from the small-scale extrusion examined in the previous section. However, the yellowing of pure polymer in production scale extrusion (Figure 12) is in a significantly lower range than for the co-processing of polymer and drug (Figure 8), although the material temperatures were partially just as, or even higher than in small scale extrusion. Furthermore, the yellowing just slightly increased with the temperature. More precisely, the yellowing varied in the range of 10% over the assessed melt temperatures from 180 to 195 °C which was not distinguishable by the human eye. Accordingly, it is concluded that the drug has a larger impact on the yellowing and degradation of the extrudates. However, yellowing of the product can be efficiently reduced by replacing the regular PS-630 by the novel PS-630U.

## 4. Conclusions

The quality of ASD formulations is influenced by both processing methods and the choice of carrier polymer. Understanding the properties of these polymers is essential for successful formulation and optimized product performance. Therefore, two copovidone grades were compared regarding polymer degradation, drug-polymer interaction, as well as processability in HME.

This study provides a comprehensive evaluation of Plasdone™ S-630 copovidone (PS-630) and Plasdone™ S-630 ultra copovidone (PS-630U) across the formulation lifecycle, including powder flow, extrusion behavior, and in-line monitoring of drug dissolution and degradation. While PS-630U demonstrated favorable characteristics such as lower peroxide content, improved feeding behavior, and reduced yellowing during processing, these findings are specific to the model systems and conditions studied. The melt properties and drug interaction profiles were found to be similar for both copovidone grades, suggesting that no processing issues are expected when replacing PS-630 with PS-630U in comparable formulations.

It is important to note that copovidone is widely recognized as a standard polymer for HME-based ASD development. This study does not aim to rank PS-630U or PS-630 against other available polymers, nor to claim superiority beyond the scope of the evaluated parameters. Rather, the results are intended to support formulation scientists and process engineers in making informed decisions when managing transitions between excipient grades, particularly in the context of lifecycle management and manufacturing optimization.

## Figures and Tables

**Table 1:** Bulk properties of PS-630 and PS-630U (*n* = 3).

Polymer	Bulk Density $kg/m^{3}$	Tapped Density $kg/m^{3}$	Carr Index %	Angle of Repose °
PS-630	327 ± 7	409 ± 3	20.13 ± 2.20	36.28 ± 1.66
PS-630U	276 ± 3	336 ± 4	17.73 ± 1.80	29.17 ± 0.32

**Table 2:** Parameters of powder flow within the extruder barrel.

Polymer	${SFL}_{max}$ −	Slip s −
PS-630	0.0195	0.918
PS-630U	0.0285	0.858

## Data Availability

The raw data supporting the conclusions of this article will be made available upon request.
